# Prism Adaptation Treatment of Spatial Neglect: Feasibility During Inpatient Rehabilitation and Identification of Patients Most Likely to Benefit

**DOI:** 10.3389/fneur.2022.803312

**Published:** 2022-04-01

**Authors:** Robert W. Gillen, Erin Y. Harmon, Brittany Weil, Benjamin Fusco-Gessick, Paul P. Novak, A. M. Barrett

**Affiliations:** ^1^Department of Neuropsychology, Sunnyview Rehabilitation Hospital, Schenectady, NY, United States; ^2^Neurorehabilitation Institute, Sunnyview Rehabilitation Hospital, Schenectady, NY, United States; ^3^Department of Psychology, Fordham University, Bronx, NY, United States; ^4^Center for Visual and Neurocognitive Rehabilitation, Atlanta VA Health System, Atlanta, GA, United States; ^5^Neurorehabilitation Division, Emory University School of Medicine, Atlanta, GA, United States

**Keywords:** prism adaptation, spatial neglect, stroke, rehabilitation, Catherine Bergego scale

## Abstract

**Objective:**

Spatial Neglect is prevalent among stroke survivors, yet few treatments have evidence supporting efficacy. This study examines the feasibility of Prism Adaptation Treatment (PAT) within an inpatient rehabilitation facility and the degree by which PAT improves symptoms of spatial neglect and functional independence among sub-acute survivors of right hemispheric stroke.

**Design:**

In this retrospective cohort study, 37 right hemispheric stroke patients were identified as having received at least 4 PAT sessions during their inpatient stay. Spatial neglect and functional independence levels of patients in the PAT cohort were compared to a matched active control group comprised of rehabilitation patients receiving alternative therapies to address neglect admitted during the same time period.

**Results:**

Most patients received the full recommended 10 sessions of PAT (average sessions completed = 8.6). A higher percentage of severe neglect patients receiving PAT (69%) displayed clinically significant gains on FIM (≥22 points) compared to those receiving alternative treatments (6%). Patients with mild or moderate neglect in the PAT cohort did not exhibit greater benefit than controls.

**Conclusion:**

Provision of PAT for treatment of spatial neglect in right hemispheric stroke patients was feasible during the inpatient rehabilitation admission. Patients with severe neglect showed the most benefit from PAT.

**Clinical Trial Registration:**

This study was registered as a retrospective observational study on Itab Clinical Trials.gov. NCT04977219.

## Introduction

Left sided spatial neglect is a common yet potentially debilitating phenomena associated with right hemisphere stroke. Heilman defines neglect as “the failure to report, respond or orient to novel or meaningful stimuli presented to the side opposite of a brain lesion that cannot be attributed to either sensory or motor deficits” (1). A growing body of research has demonstrated the heterogeneity of the disorder both in terms of symptom constellations and underlying pathology (2–5). These differences in subtypes may have important implications both in terms of the likelihood of spatial neglect persisting (i.e., becoming chronic) as well as amenability to treatment.

Studies have demonstrated the negative impact of spatial neglect in right hemisphere stroke patients on rehabilitation outcomes (6–12) and subsequent functioning in the community. Within inpatient rehabilitation settings, patients with neglect have lower functional status at both admission and discharge and show less improvement during the admission (10, 12). Spatial neglect has also been associated with longer length of stay, greater risk of falls, and reduced likelihood of home discharge (10, 12).

Given the significant negative impact of spatial neglect, numerous treatment approaches have been employed with mixed success recently reviewed by Gammeri et al. (13) yet there is lack of consensus in terms of the gold standard of treatment (14). One common approach for treating spatial neglect has been prism adaptation treatment (PAT), which has been associated with decreasing the severity of neglect with positive impact on important functional behaviors (15–17). Despite these encouraging findings, the use of PAT is not widespread in rehabilitation settings and not all studies evaluating the treatment have shown improvement (18, 19). Even in studies showing positive effects of PAT, not all patients with spatial neglect benefit from the treatment.

Heterogeneity in the neurological structures that cause spatial neglect (4) and diversity in neglect presentation (perceptive/visuospatial, exploratory/visuomotor, and allocentric/object-centered) may in part explain the variability in results.

Other unresolved issues involve optimal timing of this intervention. While most studies have targeted patients with spatial neglect in the chronic phase of stroke recovery, there have been some studies which have investigated efficacy of PAT within the early stages of recovery with mixed results (16–22).

Early intervention would seem to have both advantages and disadvantages; while it has been hypothesized that the first 30 days post stroke may represent a critical window for neuroplastic brain recovery (23), this would also represent the time period during which other stroke related deficits (e.g., hypoarousal, decreased capacity to sustain attention)which might negatively impact participation in PAT, may be most severe.

While there is some evidence that provision of PAT during months 1–3 can be effective (20, 24) there is a paucity of evidence regarding the effectiveness of even earlier interventions. In some settings, this can be important for logistical reasons; e.g., in the United States, inpatient rehabilitation facilities provide early rehabilitation services following discharge from acute hospitals. Stroke patients are discharged from acute care an average of 5.4 days following stroke (25) and spend an average of 15 days in inpatient rehabilitation facilities (26). Approximately 70% of patients with stroke do not receive outpatient rehabilitation services following discharge (27). This period also frequently encompasses the transition from hospital to home, and even small improvements in functional independence may result in reductions in falls, hospital re-admissions, or need for supervision.

There are a number of other unanswered questions regarding PAT including both minimum necessary and optimum dosing (28). The former becomes particularly important when considering the feasibility of providing treatment within the acute rehabilitation hospitalization, where in some locations (e.g., United States), lengths of stay are shrinking. Can enough sessions of PAT be administered within the admission to have a meaningful effect?

There is also the question as to whether certain severity levels or subtypes of neglect are more apt to benefit from PAT (20, 23, 29). Research findings regarding this have been mixed; Mizuno et al. (20), found that patients with mild neglect showed greater benefit then those with more severe neglect (16). In contrast, Gossman et al. (22) found that PAT was effective in patients with severe neglect of certain subtypes (egocentric vs. allocentric). Given the nature and extent of their anatomical damage, patients with severe neglect may be more likely to experience hypo-arousal, difficulties sustaining attention, deficient spatial working memory and other deficits which negatively impact capacity to participate in PAT.

The current retrospective analysis examined the feasibility and efficacy of PAT in improving symptoms of spatial neglect and functional capacity of patients in an acute rehabilitation hospital with divergent spatial neglect treatment practices, including PAT. Given that stroke team assignment within the hospital was based on bed availability, this afforded an opportunity to compare closely matched patients, differing primarily on whether they had received PAT or an alternate therapy addressing spatial neglect.

The study also explored whether PAT increased the likelihood of patients achieving a meaningful functional improvement. This was evaluated using Benninto's determination that a FIM change of 22 points represented clinically significant (minimally clinically important difference-MICD) in stroke patients (30).

It was hypothesized that right hemisphere stroke patients with spatial neglect who received PAT would show greater improvement in neglect then those who did not and show a greater likelihood of significant functional improvement.

## Materials and Methods

### Study Design

The medical records of 524 right hemispheric stroke admissions occurring between June 2016 and September 2019 were reviewed for this study. 255 patients were excluded because they had Catherine Bergego Scale (CBS) scores of <4 on admission, or incomplete CBS assessments (defined as having <7 CBS items scored on admission or discharge). Thirty seven patients with evidence of traumatic brain injury (including subdural hemorrhage), history of brain cancer, brain metastasis, Parkinson's Disease, Alzheimer's disease, dementia, or aphasia were excluded. Two hundred and thirty two met inclusion criteria. Of these patients, 47 received PAT during their stay but only 39 completed 4 or more sessions. The latter minimum treatment session criteria was based on the findings of Goedert et al. (28). Controls were chosen from admissions occurring during the same time, also meeting inclusion criteria and receiving standard treatment of neglect (Refer to Spatial Neglect Treatments below). Patients who received only 1–3 PAT sessions were excluded as possible controls. Control patients were matched to PAT patients using total CBS and total functional independent measures (FIM) scores on admission. Patients were considered a suitable match if they were within the same severity rating classification. The average difference in CBS scores was 0.84 points and in no case did matches exceed a 4-point difference. The average difference in total FIM score at admission was 2.7 points and did not in any case exceed a 7-point difference. Matches were found for 37 patients who received prism therapy (we were not able to find a matched control for 2 patients).

### Spatial Neglect Treatments

Therapists administering PAT followed the protocol developed by the Kessler Foundation (31) and detailed in previous reports (23). PAT sessions lasted ~30 min during which patients with left-sided neglect don 20 diopters deviating their visual field to the right while aiming their finger at a series of visual targets. Patients were administered PAT as a function of treatment team preference. At the time of admission, all right hemisphere stroke patients were assigned to treatment teams based on bed availability; some stroke teams provided PAT for treatment of neglect while others used customary strategies for neglect treatment (e.g., visual scanning training, limb activation).

### Spatial Neglect Assessment

Spatial neglect is routinely assessed at this facility using the Catherine Bergego scale (CBS) within 5 days of admission, and prior to patient discharge. The CBS has been shown to have excellent internal consistency (12, 31, 32) and interrater reliability (33, 34). Assessments were completed by a combination of speech, occupational and physical therapists administering those items most closely related to the area of neglect being assessed (e.g., physical therapists administering items concerning navigation). Level of spatial neglect was ranked (0–3) on 10 items (dressing, eating, navigation, etc.). Individual item scores are summed to give a total score ranging from 0 to 30.

Only CBS assessments in which 7 or more items were scored were included. In cases where 7–9 CBS items were assessed, an adjusted score was calculated using the equation below.


$$\begin{array}{l} Adjusted\;CBS\;Score=10\;x\;\frac{Total\;Score}{Total\;number\;of\;Items\;Scored} \end{array}$$

The adjusted score was used in 40.5% of participants. Neglect severity classifications were made using adjusted CBS scores according to Azouvi et al. (32) as mild (1–10), moderate (11–20) or severe (21–29, 33, 35, 36). CBS improvement was calculated by subtracting adjusted CBS scores at discharge from CBS admission scores.

### Functional Independence Assessment

The Functional Independence Measure (FIM) (37) was used to assess functional independence on admission and discharge. The FIM is an 18-item indicator of the level of assistance required to perform basic activities of daily living and includes both motor and cognitive domains. FIM items are scored on a scale of 1–7, with a 7 reflecting a level of total independence. Total scores range from 18 to 126.

### Statistical Analysis

Categorical Variables (race, sex, prior stroke, previous living arrangements) were obtained from erehabdata.com and compared between control and PAT treated groups using Fisher's Exact Test. CBS scores and FIM scores were compared using the Mann-Whitney non-parametric tests due to the ordinal nature of the FIM scores and the non-Gaussian distribution of the CBS and FIM data.

Spearman's correlation was used to determine the variables associated with CBS improvement, FIM improvement and length of stay. Spearman's ρ was reported to indicate the strength and association of these relationships.

All statistical analysis was performed using IBM SPSS Statistics (V26). *P* < 0.05 was considered statistically significant.

### Ethical Considerations

This retrospective analysis was determined to meet criteria for an exempt study by the Institutional Review Board (IRB) based on the criteria put forth by the federal regulations as defined in 45 CFR 46.101(b). A waiver of informed consent was granted.

## Results

Right hemispheric stroke patients were admitted to an inpatient rehabilitation facility, on average, 6 days [interquartile range (IQR) 4–8] following stroke onset. Eighty three of the patients administered PAT received 4 or more sessions during their stay in the rehabilitation facility—the minimum number of sessions recommended by Goedert et al. (28). No patients received more than 10 sessions. 37 matched controls were identified for the 37 patients that received 4 or more sessions. The median number of prism sessions received by this group was 10 (IQR: 8–10). The average number of sessions was 8.6. 94.6% of patients administered prism had 6 or more sessions. Treatment was initiated an average of 15 days (IQR: 12–19) following stroke onset.

### Patient Characteristics

There was no difference in the age, sex or race of participants (Table 1). All patients had suffered a right hemispheric stroke, and hemorrhagic stroke occurred at similar percentages in both groups.

**Table 1:** Admission characteristics of patients who received standard or Prism Adaptation Treatment (PAT).

	**Standard therapy**	**PAT**
Number of Patients	37	37
	**Number of patients (%)**	**Number of patients (%)**
Sex (female)	22 (59.6%)	18 (48.6%)
White	32 (86.5%)	30 (81.1%)
African American	4 (11%)	6 (16%)
Asian	0 (<3%)	1 (<3%)
Hispanic Latino	1 (<3%)	0 (0%)
	**Median (IQR)**	**Median (IQR)**
Age	71 (60–82)	70 (60–78)
	**Number of patients (%)**	**Number of patients (%)**
Hemorrhage indicated	6 (16.2%)	8 (21.6%)
	**Median (IQR)**	**Median (IQR)**
CBS Admission Score	17 (9–22)	16 (9–21)
	**Number of patients (%)**	**Number of patients (%)**
- Mild Neglect (4–9)	11 (29.7%)	11 (29.7%)
- Mod. Neglect (11–19)	13 (35.1%)	13 (35.1%)
- Severe Neglect (20–30)	13 (35.1%)	13 (35.1%)
	**Median (IQR)**	**Median (IQR)**
Total FIM at Admission	41 (32–46)	39 (34–48)
Total FIM Motor	20 (16–25)	21 (17–25)
Total FIM Cog	19 (16–22)	19 (16–21)
	**Number of patients (%)**	**Number of patients (%)**
Hemiplegia	36 (97.3%)	36 (97.3%)
Monoplegia	1 (<3%)	1 (<3%)
Lived alone prior	8 (21.6%)	18 (48.6%)^*^
Lived home prior	37(100%)	36 (97.3%)

Mann Whitney U test was used to compare: age, Catherine Bergego Scale (CBS) Admission Score, Total Functional Independence Measure (FIM) Scores. Fisher's Exact Test was used to compare: sex, race, prior stroke. % of patients in severity category, % with hemiplegia. Monoplegia. “^*^” indicates P < 0.05.

The 37 patients in the PAT group were selected based on meeting all inclusion criteria. The 37 matched control patients were selected based on having as close as possible match to the CBS and FIM scores of the prism treated patients at admission. Table 1 shows the median and interquartile ranges of the CBS and FIM scores at admission for both groups, indicating a similar level of neglect and functional independence in both groups.

### The Effect of Prism Treatment on CBS Improvement

A Spearman's correlation was performed to determine the variables associated with CBS improvement. This was done separately for patients receiving standard therapy and patients who received PAT as part of their care (Table 2A). Neglect severity on admission was not related to CBS improvement among those receiving standard care. However, there was a positive relationship between CBS scores on admission and CBS improvement among patients who received PAT (Spearman's ρ = 0.680, *P* < 0.001, Table 2A); PAT patients with greater neglect severity showed the most improvement on the CBS. In addition, PAT patients with longer admissions also showed greater improvement on the CBS (ρ = 0.36, *p* < 0.05, Table 2A). This was not the case in patients who received standard care.

**Table 2:** Variables associated with improvement outcomes in patients who did and did not receive Prism Adaptation Treatment (PAT).

	**Standard therapy**		**PAT**	
**A.Variables associated with CBS improvement**				
	**Spearman's** **ρ**	***P*** **-value**	**Spearman's** **ρ**	***P*** **-value**
CBS ADM	0.314	0.058	0.680	<0.001^*^^*^
Total FIM ADM	0.187	0.268	0.128	0.451
Age	0.044	0.798	−0.501	<0.01^*^
LOS	0.259	0.121	0.363	<0.05^*^
**B.Variables associated with FIM improvement**				
CBS ADM	−0.394	0.05	0.177	0.287
Total FIM ADM	0.542	<0.01^*^	0.176	0.298
Age	−0.008	0.964	−0.543	<0.01^*^
LOS	0.209	0.213	0.126	0.459
**C.Variables associated with FIM change per day**				
CBS ADM	−0.432	<0.01^*^	0.115	0.498
Total FIM ADM	0.397	<0.05^*^	0.267	0.110
Age	0.083	0.624	0.395	<0.05^*^

Spearman's correlation was used to determine relationships between total Catherine Bergego Scale (CBS) scores on admission (ADM), total Functional Independence Measure (FIM) scores on admission, age and length of stay with A. CBS improvement, B. FIM improvement or C. FIM change per day. Spearman's ρ and P-values are reported. “^*^” indicates P < 0.05.

There was no relationship with total FIM scores at admission and CBS improvement for either the control or PAT group. Older age was negatively related to CBS improvement in the PAT group Spearman's (ρ = −0.501, *p* < 0.01, Table 2A).

Based on the observation that PAT patients with more severe neglect showed the most improvement on the CBS, patients were stratified into 3 categories (mild, moderate or severe), based severity of neglect on admission (36). CBS scores at discharge were compared after stratification (Table 3). Patients in the severe category showed significant benefit from prism therapy; the median CBS score at discharge for patients with severe neglect was 15, IQR: 13–17 vs. 20, IQR: 13–17 (Table 3, *p* < 0.05). The discharge scores of patients with mild or moderate neglect did not significantly differ with PAT treatment.

**Table 3:** Improvement of neglect, functional independence and other outcomes in patients who received standard therapy or standard therapy with Prism Adaptation Treatment (PAT).

	**Standard therapy**	**PAT**
**CBS admission scores**	**Median (IQR)**	**Median (IQR)**
Total (*N* = 74)	17 (9–22)	16 (9–21)
- Mild Neglect on ADM (*n =* 22)	8 (6–9)	8 (6–9)
- Moderate Neglect on ADM (*n =* 28)	17 (14–17)	16 (14–18)
- Severe Neglect on ADM (*n =* 26)	25 (21–28)	23 (21–26)
**CBS discharge scores**		
Total (*N =* 74)	9 (6–17)	12 (8–15)
- Mild Neglect on ADM	5 (3–8)	8 (5–10)
- Moderate Neglect on ADM	9 (6–17)	14 (10–15)
- Severe Neglect on ADM	18 (13–23)	15 (13–17)*
**CBS improvement**		
Total	2 (0–9)	4 (0–6)
- Mild Neglect on ADM	1 (0 to 6)	0 (−1 to 2)
- Moderate Neglect on ADM	8 (0 to 10)	4 (1 to 6)
- Severe Neglect on ADM	2 (0–13)	10 (4 to 14)
**FIM admission scores**		
Total	41 (32–46)	39 (34–48)
- Mild Neglect on ADM	45(36–53)	45 (37–50)
- Moderate Neglect on ADM	41 (31–47)	34 (28–46)
- Severe Neglect on ADM	33 (31–43)	38 (34–44)
**FIM discharge scores**		
Total	57 (43–76)	64 (43–75)
- Mild Neglect on ADM	75 (56–84)	73 (51–77)
- Moderate Neglect on ADM	59 (45–78)	60 (43–67)
- Severe Neglect on ADM	43 (39–56)	64 (43–75)*
**FIM improvement**		
Total	17 (10–28)	22 (12–31)
- Mild Neglect on ADM	24 (13–35)	20 (10–32)
- Moderate Neglect on ADM	24 (15–33)	21 (13–28)
- Severe Neglect on ADM	10 (7–17)	25 (13–32)*
**Improved by 22 points on FIM**	**Number of patients (%)**	**Number of patients (%)**
Total	16 (43.2%)	18 (48.6%)
- Mild Neglect on ADM	8 (72.7%)	5 (45.5%)
- Moderate Neglect on ADM	7 (53.8%)	6 (46.2%)
- Severe Neglect on ADM	1 (7.7%)	9 (69.2%) *
**FIM gain per day**	**Median (IQR)**	**Median (IQR)**
Total	0.88 (.66 to 1.6)	0.74 (.45–1.1)
- Mild Neglect on ADM	1.2 (.86 to 1.7)	0.71 (0.45 to 1.4)
- Moderate Neglect on ADM	0.92 (0.78 to 1.7)	0.60 (0.43 to 1.0)
- Severe Neglect on ADM	0.57 (0.35 to 1.0)	0.88 (0.56 to 1.2)
**Improved 1 point or more per day on FIM**	**Number of patients (%)**	**Number of patients (%)**
Total	17 (45.9%)	14 (37.8%)
- Mild Neglect on ADM	8 (72.3%)	4 (36.4%)
- Moderate Neglect on ADM	6 (46.2%)	4 (30.8%)
- Severe Neglect on ADM	3 (23.1%)	6 (46.2%)
**Discharged to home**	**Number of patients (%)**	**Number of patients (%)**
Total	18 (48.6%)	12 (32.4%)
- Mild Neglect on ADM	10 (90.9%)	4 (36.4%)*
- Moderate Neglect on ADM	4 (25.7%)	4 (35.7%)
- Severe Neglect on ADM	4 (31%)	4 (31%)
**Length of stay**	**Median (IQR)**	**Median (IQR)**
Total	19 (13–23)	26 (20–29)*
- Mild Neglect on ADM	21 (12–28)	26 (21–28)
- Moderate Neglect on ADM	19 (12–23)	28 (19–36)*
- Severe Neglect on ADM	16 (13–25)	25 (20–31)*

Catherine Bergego Scale (CBS) improvement and Functional Independence Measure (FIM) gain were calculated as the difference in discharge and admission (ADM) scores for each patient. Mann Whitney U tests were used to determine statistical differences in CBS improvement, FIM gain, FIM gain per day and LOS in patients who received standard or prism adaptation therapy. Differences in the percentages of patients improving on the CBS or FIM scales, and the percentages of patients that were discharged to home were compared using Fisher's Exact Tests. “^*^” indicates P < 0.05.

### The Effect of Prism on FIM

To determine whether PAT can positively impact the capacity to perform activities of daily living, we looked to see if patients who received prism therapy showed greater improvement on their FIM scores. Consistent with previous reports (10, 12), a Spearman's analysis found higher CBS admission scores in the standard care group associated with poorer FIM gain (Table 2B, ρ = −0.394, *p* = 0.05) and FIM change per day (Table 2C, ρ = −0.432, *P* < 0.01). However, FIM gain and FIM change per day was not associated with CBS scores in the PAT group (Table 2B, ρ = 0.177, *p* = 0.287; Table 2C, ρ = 0.115, *P* = 0.498). In agreement with previous research, there was a positive relationship between FIM scores at admission and FIM gain (0.54, *p* < 0.01) in the standard care group, indicating that patients with higher levels of functional independence at admission usually make greater gains. However, this was not observed in PAT patients (Table 2B, ρ = 0.176, *p* = 0.298); prism training allowed patients with lower FIM scores at admission to achieve equal levels of improvement as those with higher scores.

Age also had a negative relationship with FIM improvement in the group that received prism therapy (Table 2B, ρ = −0.543, *P* < 0.01); older patients improved less with PAT then younger patients. Age was not associated with FIM improvement in the control group. Length of stay was not associated with FIM gain in either group.

FIM improvement was examined after stratification by level of neglect on admission. Patients with severe neglect made significantly greater improvements on FIM if they had received PAT (25 points, IQR: 13–32) then if they did not (10 points, IQR: 7–17) (Table 3, *p* < 0.05). The median FIM score at discharge was 64 for severe patients who received PAT, compared to 43 in patients who did not (Table 3, *p* < 0.05). PAT did not significantly improve FIM gain for patients with moderate or mild neglect (Table 3).

To evaluate significance of FIM change, Beninato's minimal clinically important difference (MCID) of ≥22 point improvement was utilized to dichotomize patients as achieving or not achieving a significant functional gain during their admission (30). Table 3 shows that patients with severe neglect more frequently achieved the MCID if they received prism therapy (62%) vs. as compared to 8% if they had not (Fisher's Exact Test, *P* < 0.05).

FIM efficiency (FIM change per day) was compared between patients who did and did not receive PAT as part of their inpatient therapy. Patients with severe neglect showed greater benefit from PAT (0.88 points per day) as compared to those that did not (0.57 points per day, but this did not reach statistical significance (Table 3). Patients with mild or moderate neglect tended to have lower FIM efficiency than those with severe neglect, but this did not reach statistical significance.

### Length of Stay

Patients who received PAT as part of their rehabilitation had significantly longer LOS as compared to the patients who did not. The median LOS for patients with severe neglect receiving PAT was 25 days vs. 16 days for control patients (Table 3, *P* < 0.05). Patients with moderate neglect were found to have significantly longer hospital stays as well if they received PAT (28 vs. 19 days, Table 3, *P* < 0.05).

## Discussion

While PAT has emerged as a promising treatment method for patients with spatial neglect, much remains unknown regarding this approach. This retrospective analysis capitalized on the variable use of PAT within an acute rehabilitation facility. On average, PAT was initiated 15 days post stroke. Most studies investigating PAT during inpatient rehabilitation initiated treatment more than 30 days post stroke (18–20, 23).

With shrinking hospitalization lengths the ability to provide the number of PAT sessions necessary to affect change during increasingly short admissions is critical. In the current study, 56% of the patients in the PAT treatment group received the full 10 session protocol with 62.2% receiving 8–10 sessions. On average, patients receiving PAT received 8.6 sessions. Administration of either the recommended full protocol or a close proximity was possible in most cases during the inpatient admission. Given data suggesting that as many as 70% of stroke patients discharged from inpatient rehabilitation settings do not receive any additional rehabilitation services (27), the ability to provide potentially effective treatments within the inpatient setting is noteworthy.

When considering the entire sample, there was not a significant difference in CBS improvement between patients receiving PAT and those who did not. However, when stratified according to neglect severity, a different picture emerged; whereas patients with mild or moderate neglect did not differ from controls in terms CBS discharge scores, patients with severe neglect had significantly lower scores than those receiving standard care (PAT-−15; Control-−18). Given the fact that more severe levels of neglect are associated with more significant negative outcomes during inpatient admissions (12) an intervention which is most effective with more severe forms of the disorder would seem highly advantageous.

The current findings differ from those reported by Ten Brink et al., who did not find differences between patients receiving PAT and those receiving a sham treatment during rehabilitation, even among patients they described as having moderate to severe neglect (19). There were, however, important differences between the two studies; treatment in the current study was initiated much earlier (15 days vs. 41 days) and patients in the present study had more severe spatial neglect.

Recent research classifying distinct subtypes of spatial neglect (2, 4) and identification of specific cortical/subcortical locations and neural networks (2, 3, 5) implicated in spatial neglect underscores the complexity of the phenomena and may contribute to an understanding of the factors underlying treatment response. For example, severe neglect is often associated with more extensive damage, involving multiple areas of the brain and as a result, more likely to involve subcortical paraventricular white matter tracts, impairing communication between the frontal and parietal regions (4). This may lead to motor intentional and visuospatial presentations of neglect, respectively. Studies suggest that patients with aiming/motor intentional spatial neglect may experience the most benefit from PAT (23, 29, 38). While lesion location and aiming spatial neglect classification was not available in the current sample, given the overall severity of the sample, it is possible that the patients with severe neglect in this study had motor-intentional spatial neglect, possibly explaining their tendency to benefit more from treatment.

Greater understanding of the pathophysiological subtypes of spatial neglect, as well as the distinct spatial attentional networks which underlie these subtypes may eventually allow better prediction of PAT responders and non-responders. For example, research suggests that patients with allocentric (object-centered) are less likely to benefit from PAT as compared to patients with egocentric neglect (22). Research by Corbetta et al. (39, 40) and Chica (41) has identified differences in attentional network impairment (bottom-up vs. top-down) in subtypes of neglect (39–41). Given the fact that PAT has been considered a “bottom up” intervention, integrity of this attentional network may be critical to treatment response.

The findings from the current study differ from several previous studies regarding the ability of patients with severe neglect to benefit from PAT. For example, Vilimovsky et al. (18) reported that patients with “moderate to severe spatial neglect receiving sham treatment improved as much on the CBS and paper and pencil measures of neglect as patients receiving PAT within an inpatient rehabilitation setting. Of note, the severity of neglect in of the PAT cohort was less than the present study. Treatment was also initiated much later (76 days post stroke/injury). This difference in spatial neglect severity may be critical if it is the most severely impaired patients that benefit from PAT.

The benefit of PAT for patients with severe neglect has been questioned, particularly due to low levels of arousal and difficulty in sustaining attention that often accompanies the neglect in these patients. Mizuno et al. (20), found that patients with mild neglect showed greater benefit then those with more severe neglect. Interestingly, our findings suggest that patients with severe neglect can benefit if attention can be sustained for prism treatments.

Perhaps the most important issue is whether amelioration of spatial neglect as measured by CBS improvement translates into significant functional gain (15, 19). Chen et al. (17) found that patients receiving PAT during inpatient rehabilitation (admissions occurring a median of 7.5 days post stroke) showed greater improvements in FIM compared to matched controls, though the magnitude of improvement was modest (3.1 points) and the intervention did not increase the number of patients achieving the MCID. Chen did not indicate the number of patients in the sample with severe neglect, but did report a lower median CBS score (10 vs. 16 in the present study).

In the current study, the negative impact of neglect on functional gain was mitigated by PAT in patients with severe neglect; Patients with severe neglect who received PAT were discharged with significantly higher FIM scores (DC FIM = 64) as opposed to those who did not (DC FIM = 43). The median FIM improvement score in severe neglect patients who received PAT was 25 points, while in those that did not it was 10 points.

A similar pattern was evident when considering whether treatment effects are clinically meaningful; 69% of patients with severe neglect receiving PAT made a clinically significant improvement compared to only 8% of those who received standard care.

Provision of PAT was associated with longer lengths of stay, particularly in patients with moderate or severe neglect. There may be several reasons for this: By chance, a higher percentage of PAT patients lived alone prior to their stroke than those receiving standard care (49% vs. 22%), complicating planning for a safe discharge. Length of stay may have been extended for some patients receiving PAT because they were demonstrating evidence of progress. With increasing pressures from third party payers to shorten hospital stays, patients who fail to demonstrate measurable functional gains are more apt to be discharged to settings where treatment is less intensive. The presence of measurable functional progress in PAT patients may have been a factor in extending their length of stay.

Despite evidence that shortened lengths of stay are associated with worse functional outcomes (42, 43), financial pressures to shorten lengths of stay have increased. In the current case, the investment in rehabilitative care, particularly as pertains use of PAT in patients with severe spatial neglect, appears to have had a significant benefit with regards to functional capacity.

Consistent with previous studies, the current study found that not all patients administered PAT benefited beyond what would be expected in this setting. Rehabilitation inpatients receive many forms of therapy during their stay (e.g., visual scanning training, limb activation) that may reduce symptoms of neglect and improve functional independence. Vilimovsky et al. (18) hypothesized in cases where spatial neglect is less severe, alternative treatments may be sufficient to ameliorate neglect. Research suggests that functional independence measures are less affected by mild spatial neglect (12), such that treatment strategies may have little impact on these measures.

One unexpected finding was that older patients were less apt to benefit from PAT. The reasons for this are not clear and the finding did not apply to all older patients; 2 out of 3 patients with severe neglect older than 71 exceeded a critical 22-point threshold for FIM MCID.

### Study Limitations

The current study has several limitations: Our modest sample size may have limited our power to detect improvements in patients with mild and moderate spatial neglect. Replication of the study with a larger sample size and blinded ratings of neglect and functional improvement is recommended. This would allow use of multiple regression models to account for effects of demographic covariates and neglect severity.

This study is limited by its retrospective nature. While not a randomized controlled trial, the patients who did not receive PAT treatment represented a closely matched naturally occurring control group. Assignment to the stroke teams was based solely on bed availability and utilization of PAT by two of the four treatment teams was a function of provider preference. Concerns regarding other unintended differences was addressed by matching treatment and control patients by both CBS and FIM admission score.

The retrospective, clinical nature of the sample did not allow inclusion of imaging, which would have allowed further characterization of the sample in terms of lesion location. Such information would have been informative in attempting to identify pathophysiologic subsets of spatial neglect. Ultimately, data of this type may be extremely useful in identifying patients most likely to benefit from PAT or other forms of treatment.

Similarly, reliance on a functionally based measure of neglect (CBS) limited our ability to differentiate patients as per various subtypes (e.g., Egocentric vs. allocentric, aiming/motor-intentional vs. perceptual/attentional).

As discussed above, patients receiving PAT also had longer lengths of stay, which in turn resulted in these patients receiving more treatment. While this raises the possibility that it was the additional treatment *per se* as opposed to the PAT that resulted in the improved outcomes in the severe neglect patients, neither the longer admission nor the additional treatment time improved outcomes in the mild or moderate groups. While longer lengths of stay may have contributed to improved FIM outcomes, PAT patients with mild and moderate neglect did not show a comparable level of FIM improvement despite also receiving additional treatment time. The case can be made that if it requires additional treatment or a longer length of stay to affect a significant reduction in severe neglect, then this represents a worthwhile investment of time and resources.

Another limitation of the current study is the lack of follow-up data. It is unknown whether the treatment gains of the severely impaired PAT group persisted over time, nor whether the mild-moderate PAT groups eventually exhibited benefits following discharge. Several studies have demonstrated persistence of treatment effects over time (20, 44, 45). Unfortunately, without external funding, it was not possible to follow-up patients in the current study.

The current findings do not shed further light on whether certain subtypes of neglect are more likely to be respond positively to PAT. Goedert et al., identified a perceptual-attentional and motor exploratory subtypes of neglect using the CBS (29, 46) and suggested that patients with the motor exploratory subtype may be more likely to benefit from PAT. The current data will be analyzed to determine if a similar pattern was present in current responders/non-responders.

## Conclusion

Prism adaptation treatment was administered to 37 right hemisphere stroke patients during their acute rehabilitation admission. With treatment initiated ~2 weeks following stroke, this represented one of the earliest PAT interventions in the research literature. In a majority of cases (56%), the full recommended 10 session dosage was administered, while a close proximity was achieved in most others (average sessions completed = 8.6). When compared to matched controls receiving standard treatment, PAT had minimal impact in terms of either neglect or FIM improvement in patients with mild or moderate neglect, but a significant impact on patients with severe neglect. Patients with severe neglect receiving PAT showed greater improvement on both the CBS and FIM. A higher percentage of severe neglect patients receiving PAT (69%) displayed clinically significant gains on the FIM ≥ 22 points) compared to those receiving standard care (8%).

As stated by Chen et al. (17), there is great need for studies of PAT in real world settings “where patients and clinical practices vary more than in a well-controlled research context”. By capitalizing on the fact that some treatment teams in the current setting, utilized PAT while others utilized more conventional methods, the current study represents the impact of this intervention in a “real world setting.” While the findings are clearly preliminary and require replication with a larger sample, inclusion of follow-up measures, and matching of amount of treatment received, they do suggest considerable benefit from PAT in right hemisphere stroke patients with severe neglect both in terms of reducing the severity of neglect and improving functional outcomes. Given the fact that more severe neglect has the most deleterious impact on functional outcomes, the findings that more severe neglect may be particularly amenable to prism adaptation treatment is potentially important.

## Data Availability Statement

The raw data supporting the conclusions of this article will be made available by the authors, without undue reservation.

## Ethics Statement

The studies involving human participants were reviewed and approved by St. Peter's Health Partners Institutional Review Board. Written informed consent for participation was not required for this study in accordance with the national legislation and the institutional requirements.

## Author Contributions

RG, BW, BF-G, and PN contributed to conception and design of the study. EH organized the database and performed the statistical analysis. RG wrote the first draft of the manuscript. EH, PN, and AB wrote sections of the manuscript. All authors contributed to manuscript revision, read, and approved the submitted version.

## Funding

Publication fees will be provided by James A. Eddy Research Institute at Sunnyview Rehabilitation Hospital.

## Conflict of Interest

The authors declare that the research was conducted in the absence of any commercial or financial relationships that could be construed as a potential conflict of interest.

## Publisher's Note

All claims expressed in this article are solely those of the authors and do not necessarily represent those of their affiliated organizations, or those of the publisher, the editors and the reviewers. Any product that may be evaluated in this article, or claim that may be made by its manufacturer, is not guaranteed or endorsed by the publisher.
